# The pursuit of radiology training in times of a pandemic

**DOI:** 10.1259/bjro.20200039

**Published:** 2020-09-07

**Authors:** Anum Pervez, Fiona McCurdie, Daniel Poon

**Affiliations:** 1 Department of Clinical Radiology, Guy’s and St Thomas' NHS Foundation Trust, Westminster Bridge Road, London, UK

## Abstract

The Coronavirus-19 (COVID-19) pandemic has been the greatest challenge faced by the National Health Service (NHS) in its lifetime. The crisis has seen the disruption of many long-held institutions, most critically of which is specialty training. In this article, we discuss the impact of the pandemic on Radiology training in the UK. We explore the methods that have been used to combat these difficulties and suggest workable solutions. As technology platforms become ever more integral to our daily clinical routines, we discuss how these offer a new approach to training. We argue that, of all the medical disciplines, radiologists are best placed to design and implement technology-based training, and lead other specialties in doing so. Whilst the upheaval of traditional approaches to education is a challenge, we propose that this departure from the norm offers exciting opportunities for improvement.

## Introduction

On December 31st 2019, the World Health Organisation was informed of a cluster of pneumonia cases in Wuhan, China. As of August 19th, 2020, over 22.1 million cases have been recorded worldwide, with more than 781,000 deaths.^1^ The novel coronavirus, COVID-19, has halted the world in its tracks. For healthcare workers in the UK, and across the globe, personal and professional roles have had to rapidly change. With redeployment of staff, changes to service provision and social distancing, daily activities have been re-prioritised, at the expense of specialty training. Whilst we look with trepidation to the coming winter, and await the threatened ‘second peak’, it is an important moment to take stock. We reflect on the impact COVID-19 has had on Radiology training and explore how to move forward, seeking opportunities for improvement in this time of crisis.

## Challenges in delivering radiology training

**Figure 1.**
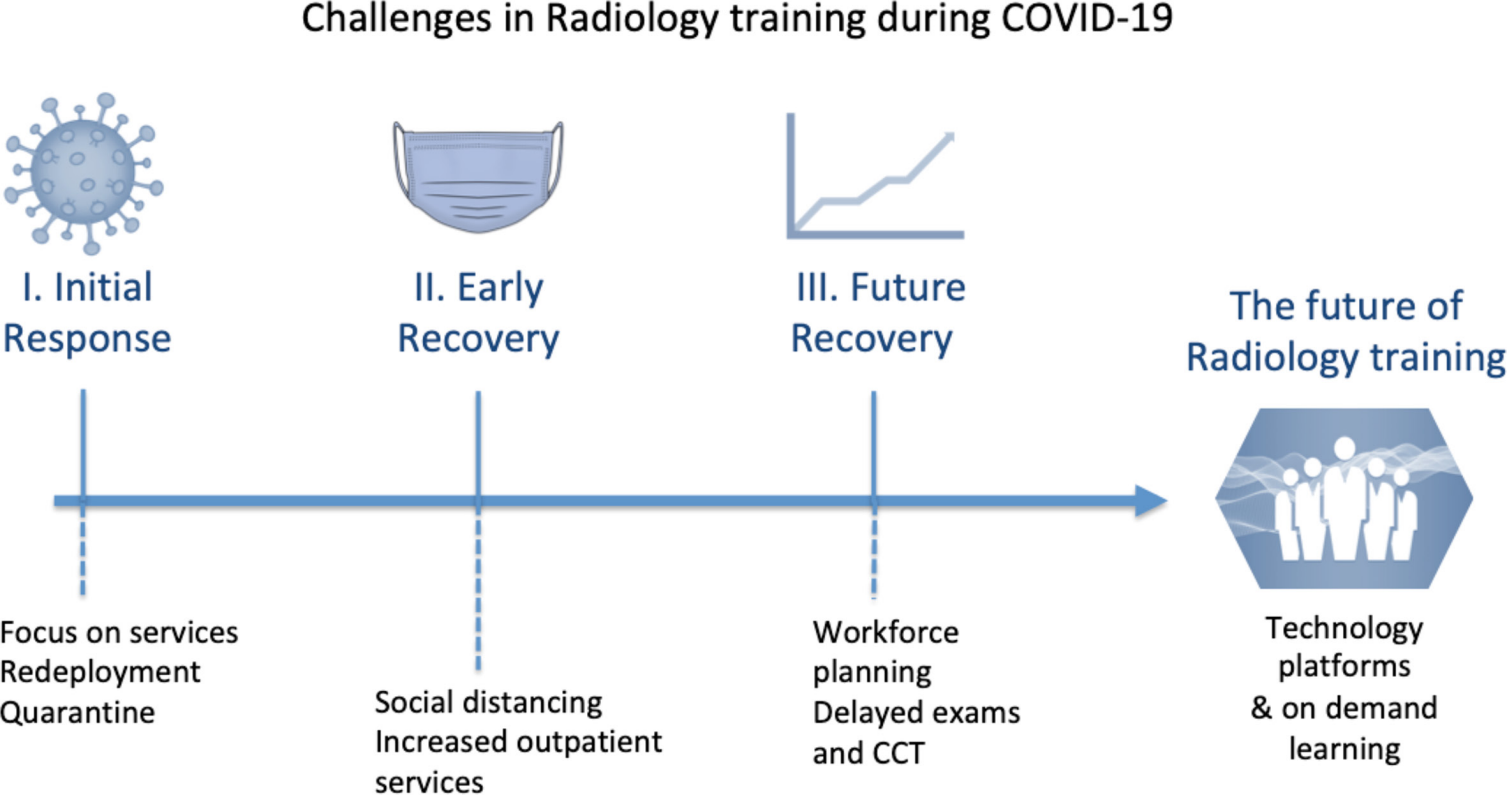
Challenges in Radiology training^2^

To explore the impact of COVID-19 on training, we break it down into three phases (Figure 1) and discuss the different challenges faced at each point in time:

### Phase I: Initial response

As the magnitude of the COVID-19 pandemic became increasingly apparent, and hospitals issued major incident warnings, Radiology departments across the country had to reconfigure the delivery of their services to match new, unprecedented demands.This put an emphasis on prompt reporting, clearing of backlogs and redistribution of the general workload. Training in this Phase came to a complete standstill. All upcoming FRCR (Fellow of the Royal College of Radiologists) examinations were cancelled, rotations were paused and trainees were redeployed to intensive care, emergency departments and medical wards. The London School of Training reported around two-thirds of ST1 and ST2 clinical radiology trainees had been redeployed, with a variable number of ST3s. These trainees went entirely without training during this period, which for some lasted months.

For those trainees left behind, new rotas were designed to ensure demand for services could be met, whilst also limiting the numbers travelling to and spending ‘non-essential’ time at work. As routine outpatient work stopped, the volume and range of reporting opportunities plummeted. This particularly impacted practical training in modalities such as ultrasound, fluoroscopy and interventional radiology.

Another challenge, not unique to radiology trainees, was the increasing rates of staff illness. Symptomatic staff members, or those living with symptomatic individuals, required adequate time off in self-isolation. Likewise those who fell into the high-risk category needed to shield for prolonged periods. During this phase, there was little infrastructure in place to access reporting remotely, participate in virtual tutorials or attend multidisciplinary meetings (MDMs) and hence this cohort of trainees were completely without any real-time interaction with the specialty.

### Phase II: Early recovery

Several months on from the initial lockdown, we are now in the Early Recovery phase, grappling with the consequences of the Initial Phase and its impact on training. Social distancing measures and a displaced workforce have meant that face-to-face teaching, both in formalised tutorials and during chance work-place encounters, have near-vanished. This has led to a loss of real-time feedback and limited the fostering of the trainee–trainer relationship, both of which are crucial to trainee development and their academic support.^3^

Service demands, in the wake of the Initial Phase, have changed the volume and nature of work. As more routine services are reintroduced, and the backlog is surmounted, shift patterns have been adjusted to allow for extra out of hours lists to be implemented. This has meant that trainees spend more time providing acute services, limiting supervised training sessions.

### Phase III: Future Recovery

As we look ahead to the next phase, Future Recovery, we consider this disruption to training and what impact it will have on the workforce. There is not a clear solution as to how to reclaim lost training time without extending length of training. This is particularly challenging for those senior trainees approaching their Completion of Certificate of Training (CCT) and those who have been unable to progress due to delayed FRCR Exams. According to the 2019 Clinical Radiology UK workforce census, there are a total of 1,636 specialty trainees. Pre-COVID, we were understaffed by 1,876 radiologists (33% of the workforce needed), which was due to rise to 3,331 (43%) by 2024.^4^ The RCR has stated that it is working with the GMC, national training organisation and heads of schools to minimise disruption to trainee progression,^5^ although there is no doubt that an already stretched workforce is about to be stretched further.

## An oppurtunity for improvement

COVID-19 has thrown many aspects of training into question, and as yet there feels to be very little in the way of answers. Whilst this is unsettling, it should be seen as an opportunity for improvement.

Traditionally, Radiology training has been delivered through case-based teaching, through reporting sessions and didactic lectures.^6,7^ The pandemic has initiated a paradigm shift away from the classroom and towards a digital, remotely accessible learning environment. Radiology, of all the medical disciplines, is best placed to welcome and drive such a shift. Radiology practice is already technology-centric, our patients are mostly encountered on a computer screen and the archives of our work are stored as an electronic resource. We are extremely well-suited to shifting classroom, case-based approaches to an entirely digital process. Moreover, we would be suited to taking a leadership role in setting up and implementing these systems for our clinical colleagues.

Whilst unable to offer a simple solution to this very complex problem, here we share some reflections on what we consider to have worked well in our department, and suggest how this could pave the way to more robust methods of training:

### “Mute your Mic”

There has been an explosion in video-conferencing platforms during the pandemic. The new ‘normal’ seems to be a constant deluge of meeting links and malfunctioning (or unmuted) microphones. Whilst these frustrations are unavoidable, there is tremendous scope for such platforms in training. Choice of which to use is likely designed, in part, by the local IT policy’s approval, but consideration should be made as to which one to use for the specific needs of training. One of the serious pitfalls of video-conference teaching is the lack of attendee engagement. We have found that there are excellent features that promote interactive learning, such as the ‘Share Control’ function in Microsoft Teams (Washington), which allows the presenter to hand over control of their screen to a trainee, who can then scroll through images. The chat function, available on all platforms, should be utilised with a nominated attendee monitoring the chat activity whilst the presenter focuses on teaching. Another strategy to encourage participation is the use of quiz-based platforms such as Kahoot! (Norway) and Poll Everywhere (San Francisco) which promote active engagement whilst introducing a competitive element to the session, if desired. It is worth noting although that these do require pre-preparation by the trainer.

Another frustration arises with loading large datasets which slow transmission and lead to jolting or blurred images. Simple alterations can improve this, for example when using PACS to present cases, choosing the thicker slice images, which allows a smoother image transfer. Choice of which screen to ‘share’ also impacts image quality, with split or single, portrait screens leading to smaller, blurrier images on the trainee’s screen.

### Radiology ‘On-Demand’

Like so many aspects of modern life – Netflix, next day delivery, Uber – we expect to have what we need available on hand straight away. Whilst formalised sessions are crucial to training, an On-Demand learning platform is an excellent addition. This allows those who have missed training to ‘catch up’ in their own personal time. We are already accustomed to some great national online eLearning platforms, such as the RCR’s R-ITI (Radiology-Integrated Training Initiative) modules,^8^ but these should be offered at a local level. Teaching files, a staple in radiology training, have traditionally allowed trainees to gain exposure by reviewing a wide range number of normal and abnormal cases.^9,10^ As discussed, loss of training time and change to service provision have led to a lack of depth and breadth of training. With some simple organisation, these collections of cases can be collected and disseminated across the trainee body to be utilised by trainees at will, or as an adjunct to a specific teaching session and hence engender a ‘Flipped Classroom approach’.^11^ These can also be employed as a self-audit, where trainees report and then subsequently mark themselves against the positive and negative findings or formalised into assessments.

## Summary

COVID-19 has irrevocably changed the face of Radiology training. Despite ongoing disruption, we should exploit this as an opportunity for meaningful change. There is ample scope to continue to deliver high standards of training with the utilisation of new technological platforms and the repurposing of existing ones. We argue that, as Radiologists, we are best suited to maximise these new opportunities and lead the way in updating and improving specialty training in the UK.
